# Induced Pluripotent Stem Cells: Reprogramming Platforms and Applications in Cell Replacement Therapy

**DOI:** 10.1089/biores.2019.0046

**Published:** 2020-04-28

**Authors:** Akram Al Abbar, Siew Ching Ngai, Nadine Nograles, Suleiman Yusuf Alhaji, Syahril Abdullah

**Affiliations:** ^1^ Medical Genetics Laboratory, Department of Biomedical Sciences, Faculty of Medicine and Health Sciences, Universiti Putra Malaysia, Serdang, Malaysia.; ^2^ School of Biosciences, Faculty of Science and Engineering, University of Nottingham Malaysia, Semenyih, Malaysia.; ^3^ Newcastle University Medicine Malaysia, Educity, Iskandar Puteri, Johor, Malaysia.; ^4^ UPM-MAKNA Cancer Research Laboratory, Institute of Bioscience, Universiti Putra Malaysia, Serdang, Malaysia.

**Keywords:** clinical applications, gene editing, iPSCs, OSKM, reprogramming, viral and nonviral vectors

## Abstract

The generation of induced pluripotent stem cells (iPSCs) from differentiated mature cells is one of the most promising technologies in the field of regenerative medicine. The ability to generate patient-specific iPSCs offers an invaluable reservoir of pluripotent cells, which could be genetically engineered and differentiated into target cells to treat various genetic and degenerative diseases once transplanted, hence counteracting the risk of graft versus host disease. In this context, we review the scientific research streams that lead to the emergence of iPSCs, the roles of reprogramming factors in reprogramming to pluripotency, and the reprogramming strategies. As iPSCs serve tremendous correction potentials for various diseases, we highlight the successes and challenges of iPSCs in cell replacement therapy and the synergy of iPSCs and *clustered regularly interspaced short palindromic repeats* (CRISPR)/Cas9 gene editing tools in therapeutics research.

## Introduction

Human embryonic stem cells (ESCs) are derived from the inner cell mass of a developing embryo at the blastocyst stage. These cells are pluripotent, that is, they have an indefinite ability to self-renew while maintaining the potential to differentiate into all cell types.^1^ ESCs offer tremendous potential applications in biomedical research and regenerative medicine, opening new avenues for therapeutic strategies aimed at cell replacement in degenerative, traumatic, and ischemic disorders.^2^ However, human ESC-related research is ethically controversial because it involves the destruction of human embryo. The ethical and legislative debates revolving around the use of human embryo in research have been circumvented by the advancements in nuclear reprogramming.

**FIG. 1.**
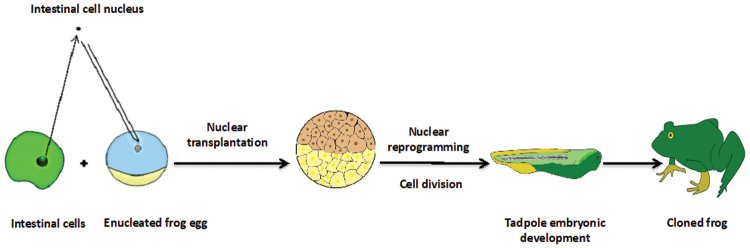
Nuclear reprogramming strategy. The nucleus of a differentiated cell is transplanted into an enucleated egg in meiotic metaphase by nuclear transfer. The transplanted genome is reprogrammed into a pluripotent state, whereby the egg undergoes cell division and a cloned animal is produced.

It was initially thought that the genome of a mature cell is everlastingly locked in a somatic state and unable to revert into a fully ESC-like state.^3^ However, Sir John B. Gurdon entirely altered this paradigm by producing a fully functional tadpole from an unfertilized egg containing a nucleus from a differentiated intestinal epithelium cell of a mature frog (Fig. 1).^4,5^ More than 30 years later, Dolly the sheep was cloned from an adult somatic cell using nuclear transfer technology.^6^ These momentous findings concluded that differentiated cells still retain the genetic memory that is important for an organism's development and that oocytes contain factors that can reprogram the mature cell's nuclei.^7^

The conservation of genome during development serves as a basis of principle for nuclear reprogramming. However, little is known about this process. It was hypothesized that the factors that play important roles in the maintenance of ESC identity also play pivotal roles in the induction of pluripotency in the somatic cells.^8^ Extensive research has been conducted in identifying these factors. Takahashi and Yamanaka^9^ were the first to demonstrate that the pluripotent stem cells could be induced from the adult fibroblasts by introducing four transcription factors, octamer-binding transcription factor 3/4 (Oct3/4), SRY (sex determining region Y)-box 2 (Sox2), Krüppel-like factor 4 (Klf4), and cellular-Myelocytomatosis (c-Myc) (OSKM).

This review discusses the scientific framework that led to the reprogramming of induced pluripotent stem cells (iPSCs), the roles of the OSKM in reprogramming the mature differentiated cells into iPSCs, and the benefits and drawbacks of the reprogramming strategies. In addition, the potential applications of iPSCs in cell replacement therapy and the synergy of iPSCs and clustered regularly interspaced short palindromic repeats (CRISPR)/Cas9 gene editing tool in therapeutics research are also reviewed.

## Induced Pluripotent Stem Cells

**FIG. 2.**
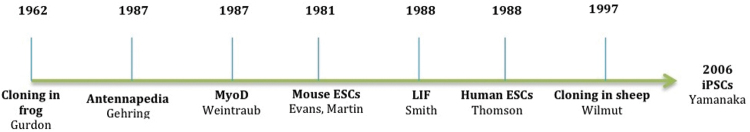
Merger of three scientific research streams that facilitates the development of iPSCs. The first stream was initialized when Gurdon produced tadpoles from an unfertilized egg using a nucleus from frog intestinal cell in 1962. With more than three decades of research using the discovery of “master” transcription factors in the second stream and the research involving ESCs in the third stream, Wilmut's group demonstrated the first birth of live mammal created by nuclear transfer technology in 1997. Subsequently, Takahashi and Yamanaka reported the generation of iPSCs from somatic cells by transduction of four transcription factors in 2006. ESCs, embryonic stem cells; iPSCs, induced pluripotent stem cells.

**FIG. 3.**
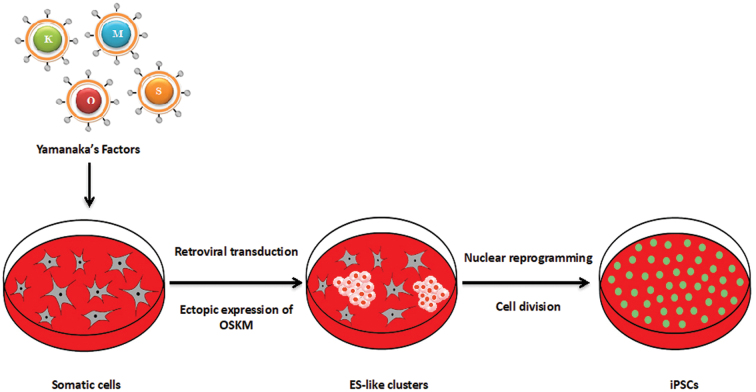
Nuclear reprogramming strategy. Ectopic expression of the four defined transcription factors associated with pluripotency (Oct4, Sox2, Klf4, and c-Myc) reverses the unipotency state into a pluripotency state. c-Myc, cellular-Myelocytomatosis; Klf4, Krüppel-like factor 4; Oct4, octamer-binding transcription factor 4; Sox2, SRY (sex determining region Y)-box 2.

The depth of Yamanaka's perception through the discoveries in somatic cell nuclear transfer,^10^ cellular fusion,^11^ ESC research,^1,12^ and understanding of pluripotency related transcription factors^13,14^ (Fig. 2) has led to the landmark discovery in stem cell research. This major breakthrough was the demonstration that ectopic expression of cellular transcription factors by retroviral vector transduction in mouse fibroblasts was sufficient to reverse a somatic cell into a pluripotent-like state (Fig. 3).

The nuclear reprogramming involves the transduction of four transcription factors—Oct4, Sox2, Klf4, and c-Myc (OSKM) —into somatic cells that led to the generation of iPSCs.^8^ In astonishment to the pioneering report of iPSCs, scientists quickly tried to reproduce and extend the work. For example, Yu et al.^15^ used Thomason's cluster consisting of a slightly different combination of transcription factors (Lin28, Nanog, Oct4, Sox2) to reprogram human embryonic, neonatal, and adult fibroblasts into iPSCs, Hanna et al.^16^ cured sickle cell anemic mice by autologous iPSC therapy, and Stadtfeld and Hochedlinger^17^ cloned mouse iPSCs.

Several findings have demonstrated that iPSCs can be differentiated into all kinds of tissue in mice, including cardiovascular and hematopoietic lineages,^18^ sperm,^19,20^ cardiomyocytes (CMs),^21^ and retinal cells.^22^ In addition, Lowry et al.^23^ reported that the human cells could also be successfully rewound into stem cell-like state. A group led by Clive Svendsen generated iPSCs from a young boy with spinal muscular atrophy and, subsequently, differentiated the iPSCs into neurons.^24^ A team of scientists led by Serrano has discovered an effective way to generate iPSCs in animal model. This finding has gone a step beyond pluripotency as the *in vivo* reprogrammed cells could develop placental cells, which both standard iPSCs and ESCs could not develop.^25^

## The Roles of OSKM Transcription Factors

The transcriptional profiling analysis by whole genome sequencing reveals that hundreds of pluripotency markers are tightly correlated with ESCs. However, only three of these transcription factors, Oct4, Sox2, and Nanog, are the critical regulators in early development and maintenance of ESC identity.^26^ Somatic cell reprogramming is initiated by changes in the transcriptome and chromatin structure of differentiated state into that of a pluripotent-like state. The ability of reprogramming transcription factors to bind to pluripotency associated recognition sequence in somatic cells is mostly modulated by the changes in chromatin structure influenced by DNA methylation, histone modifications, and ATP-dependent chromatin remodeling. The reprogramming transcription factors spontaneously bind together to form an interconnected autoregulatory circuitry, triggering their own core promoter genes and cooperating with other pluripotency associated genes.^9^ The interconnected autoregulatory loop suggests that Oct4 and Sox2 play a key role in the maintenance of pluripotency^27^ and in early embryo precursor cells,^28^ respectively. In contrast, Nanog plays a paramount role for mammalian development, growth, and differentiation of blastocyst in the preimplantation embryo.^29–31^

Transcription factor-mediated reprogramming of somatic cells into pluripotency state begins with the ectopic expression of OSKM that co-occupy an extensive subset of genomic regions in closed chromatin of somatic genes in the early part of reprogramming stage.^9^ To date, no study has described the map of OSKM transcription factor binding sites and chromatin reorganization modeling for transient reprogramming in detail. Thus, a precise knowledge about how OSKM transcription factors direct the conversion of unipotent cells into pluripotent cells remains unclear.^9,17,32,33^

However, Stadtfeld and Hochedlinger^17^ reported that two transcriptional waves are elicited when pluripotency is induced. In the first transcriptional wave, c-Myc binds to a large region of somatic genome with methylated H3K4me2 and H3K4me3, which mark of open chromatin. This allows the Oct4 and Sox2 to have access to the necessary genes for reprogramming and to the enhancers and promoters of genes that determine the somatic identity of the cells. This is followed by the silencing of somatic related gene expression, which includes mesenchymal genes such as *Thy1*, *Snai1*, *Snai2*, *Zeb1*, and *Zeb2* surface markers.^9,34^ Of note, c-Myc is a well-known oncogene that seems to be directly associated with the cycle regulation of cell proliferation and biosynthetic pathways.^9^

The second transcriptional wave is more delimited to the reprogrammed cells; OSKM access the enhancers and promoters of early pluripotency-associated genes (PAG), triggering their transcription and expression. During this wave, somatic cells were enforced to alter their morphology, increase in proliferation, and undergo mesenchymal-to-epithelial transition (MET). The MET is apparently a stochastic and inefficient process due to the presence of methylated histone on pluripotency induction genes, which are responsible for closed chromatin conformations.^9^ This leads to the upregulation of epithelial genes such as *Cdh1*, *Epcam*, and *Ocln*^35^ and the establishment of the basic state of epithelial character with the formation of larger ES-like clusters. Simultaneously, the expression of pluripotency gene network is activated, including the activation of alkaline phosphatase (AP) and stage specific embryonic antigen-1 (SSEA1) for mouse system or the surface gene TRA-1-60 for human cells.

Klf4 plays contradicting roles in both phases. First, by restraining differentiated genes in the first phase, as it binds and activates epithelial genes, including E-cadherin.^36^ Second, by accelerating the essential endogenous Oct4 and Sox2 expression in the second phase, which establishes the autoregulatory loop that maintains the pluripotent state. It is widely accepted that Klf4 naturally acts in pluripotent cells by controlling cellular processes such as development, proliferation, differentiation, and apoptosis. Klf4 has a potent interaction with Oct4 and Sox2 to activate a group of transcription factors, such as Nanog, Esrrb, Klf2, Sall4, and ZFP42, and signaling pathway regulators, such as Smad1 and Stat3. This core network between numerous pluripotency transcription genes and signaling cues provides stability to the pluripotent gene expression program.^37^

The key characteristic of subsequent reprogramming phase is the activation of core pluripotency-related genes that are associated with stable pluripotent state.^35^ For instance, the loci Nanog and Sall4 are transcriptionally upregulated at the late intermediate state, while Utf1 and Sox2 are induced even later, closely mirroring the acquisition of full pluripotency expression programming.^9,33^ The cells then move toward pluripotency through the activation of p53-p21 pathway by the two following phases.^38–40^ During the early phase, apoptotic and senescence genes are activated while the p53 protein is repressed, which enhances the reprogramming process in both mice and humans.^34^ In the late phase, the reprogramming factors are silenced, and the cytoskeleton is remodeled as closely as an ES-like state, the epigenome is reset, and the core circuitry of Nanog or unidentified factors of pluripotency are activated.^9,41,42^

## The Reprogramming Vectors

**FIG. 4.**
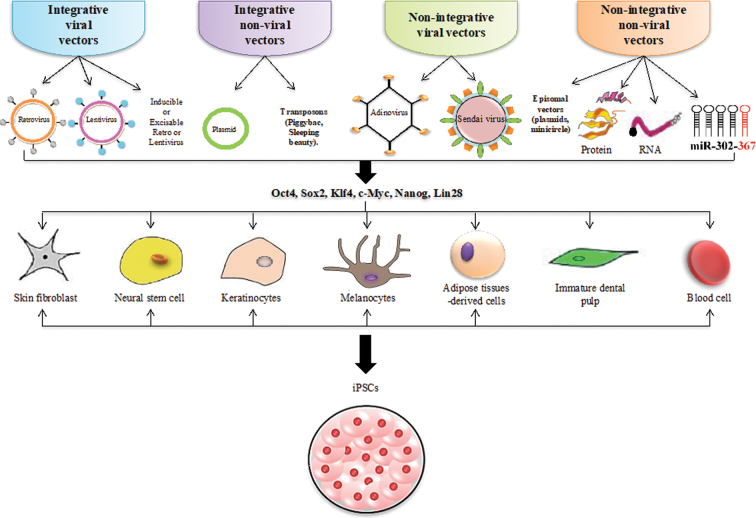
Various cell sources and transfer strategies for the generation of iPSCs. The iPSCs were initially derived from mouse embryonic and skin fibroblasts. Soon after, scientists have successfully used other somatic cells with improved reprogramming efficiency. Progress has been made in the choice of reprogramming factors, which include Yamanaka's factors, Nanog and Lin28. Integrating viral vectors like retrovirus and lentivirus were used to generate the first iPSC lines. Thereafter, nonintegrating viral vectors and plasmid systems were used. In recent times, successful reprogramming transfer strategies using recombinant and isolated proteins from ESCs have been demonstrated. Newer approaches, such as synthetic modified RNA or mRNA and miRNAs, have also been used to enhance reprogramming efficiency. miRNA, microRNA; mRNA, messenger RNA.

Following the pioneering study by Yamanaka, several groups of scientists have used different strategies to produce the iPSCs to meet the safety and quality criteria for effective therapeutic applications. These reprogramming strategies are divided into two groups: reprogramming by integrative or by nonintegrative transfer systems either by viral or nonviral methods (Fig. 4), with each strategy having its own advantages and disadvantages. In addition, no single set of reprogramming transfer system has been improved without falling prey to one of serious limitations or potential undesirable consequences.

## Reprogramming by Integrative Viral Vector Transfer System

Integrating viral vectors were used to generate the first iPSCs. Retroviral vectors have been widely used as a vehicle for gene transfer for *in vitro* and *in vivo* studies.^43^ They only provide temporal gene expression of the exogenous DNA sequence as the proviral transgene expression is silenced toward the late period of the reprogramming process^44^ due to epigenetic modifications.^45–47^ Besides, the quality of the generated iPSCs is partially impaired because of the failure to fully activate the expression of endogenous genes associated with pluripotency.^48,49^ Nonetheless, some reports indicated that the viral transgene reactivation and its residual activity in the resultant iPSCs can alter cellular developmental process and may lead to tumor formation in chimeric animals.^50,51^

Lentiviral vector (LV) is known to be more efficient than retroviral vector, because of its broad tropism.^51,52^ LV is used to reprogram many somatic cell types ranging from mouse,^44^ rat,^53^ pig,^54^ and human.^55^ LV gene delivery method still remains as the most efficient reprogramming strategy with reprogramming efficiency of 0.1–1%.^17,56,57^ Nevertheless, efforts have been made to improve the safety of this strategy.^58,59^ One of the advancements made in the design of an effective reprogramming LV is the development of a polycistronic LV, which carries all the four reprogramming factors that are linked by 2A “self-cleavage” peptide sequences in a single expression cassette. These four transcription factors are driven by a single promoter.^50,60^ The 2A “self-cleavage” peptides are 18–22 kDa amino acid derived from the aphthovirus foot-and-mouth disease virus.^61,62^ This system reduces the viral copy number integration in the transduced cells, minimizes the risk of transgene silencing, simplifies the conversion procedure, and establishes a consistent reprogramming factor stoichiometry.^63–68^ In addition, to eliminate the effects of inefficient silencing and transgene reactivation, the polycistronic viral vector has been reengineered by the introduction of excisable vector (cre/loxP system)^69,70^ and inducible (tetracycline/doxycycline inducible system) systems.^58,59,71,72^ The integrated transgene can be subsequently removed from the genome of the host cell using transient expression of Cre. However, this strategy is transduction inefficient^73^ and can lead to iPSC mutagenesis as the Cre/loxP system may leave a trace of loxP after the reprogramming process.^74–77^

## Reprogramming by Integrative Nonviral Transfer System

Due to the current limitations of integrating viral transfer system, scientists have been actively investigating other reprogramming methods such as the nonviral transfer systems, which are safer for therapeutic applications. The first successful nonviral iPSCs were produced from mature embryonic fibroblast cells transfected with two plasmid constructs; the first plasmid encodes for the c-Myc, while the second plasmid a polycistronic vector encodes the four defined reprogramming factors.^50^ These findings demonstrated that the transient overexpression of the Yamanaka factors is sufficient to induce pluripotency in somatic cells. However, the risk of integration and poor efficiency of reprogramming are the major issues.^78^ To overcome these issues, an integrated-dependent gene transfer vector was designed by incorporating the transcription factors into loxP sites of the reprogramming construct.^68,69^ However, short vector pieces can exist in the genome's cell upon excision and this may influence the cellular functions.^78^ The use of a mobile genetic element, such as piggyBac (PB) transposons, to deliver exogenous pluripotency genes is highly efficient. The remnants of this element can be completely excised from the reprogrammed cell by transient transposase expression.^67,79^ Unfortunately, human genome has endogenous PB-like transposon elements,^80^ which can cause nonspecific genomic alterations upon transgene excision.^17^ Sleeping Beauty system was introduced to overcome the PB limitations, whereby its integration frequency is lower compared to PB and the human genome has no PB-like elements.^81,82^ Unfortunately, its reprogramming efficiency is low and the use of excisable elements can lead to a risk of reintegration.^78^

## Reprogramming by Nonintegrative Viral Transfer System

Human and murine iPSCs have been successfully created using nonintegrating viral vectors such as adenovirus.^83,84^ The iPSCs obtained from these studies demonstrated no insertion of exogenous DNA in the host genome. However, the reprogramming efficiency by the current nonintegrating viral vector delivery methods is limited to 0.001%. It has been reasoned that the transient expression of OSKM was not sufficient to permit complete epigenetic remodeling.^17,80^ Nevertheless, the implementation of adenoviral method in translational medicine holds a great promise.^51^ An alternative approach is to use negative single-stranded RNA Sendai-virus (Se-V) as it is very efficient at introducing foreign genes in many types of cells and tissues. However, it is hampered by low reprogramming efficiency.^85,86^ Nevertheless, great efforts have been made to develop an improved Se-V.^87,88^ It is worth noting that Se-V has huge potential in cystic fibrosis gene therapy^89,90^ and AIDS vaccines^91^ and is applicable for human iPSC replacement therapy.^92^

## Reprogramming by Nonintegrative Nonviral Transfer System

To generate iPSCs free of vector integration into chromosomes, the pluripotency marker genes can be directly and transiently delivered into the somatic cells using cytoplasmic RNA, episomal (self-replicating and selectable vectors),^15^ or polycistronic minicircle DNA nonviral vector systems.^93^ These approaches are relatively easy to use, but the reprogramming efficiency was shown to be 5–10 times lower than LV.^51^ In contrast, the use of episomal plasmids and minicircle DNA vectors requires extensive optimization for future application.^51^

Mouse and human fibroblasts have been successfully reprogrammed by a direct transfer of the recombinant reprogramming proteins in purified forms^84^ or as total-protein extracts isolated from ESCs^94^ or transgenic HEK293 cells.^95^ However, this method is problematic as the synthesis of such proteins in large quantities is challenging, the conversion efficiency is particularly inefficient, and the cellular reprogramming process requires 8 weeks. Generation of iPSC by chemical reprogramming may work, but this process may lead to mutation, as the genome of the cell is vulnerable to DNA and histone modifications.^17,78^

To overcome these issues, the introduction of either synthetic RNA or messenger RNA (mRNA) encoding the reprogramming factors may be a powerful platform for creating integration-free pluripotent cells. Although multiple rounds of transfection may be required, these methods are relatively efficient in generating iPSCs with better safety profiles.^96,97^ To improve the reprogramming process, new approaches such as microRNAs (miRNAs) have been used to enhance the reprogramming efficiency. For instance, miR-291-3p, miR-294, and miR-295 have been used instead of c-Myc to produce homogeneous human iPSC colonies.^98^ However, the inhibition of *let-7* miRNA has enhanced c-Myc expression, whereas Lin*-28* has promoted cell-reprogramming process.^99^ Another study reported that a cluster of miRNA 302/367 was successful in reprogramming mouse and human somatic cells into iPSCs without the employment of transcription factors, despite low reprogramming efficiency.^100^

## Feeder-Free and Defined Conditioned Culture Medium

**FIG. 5.**
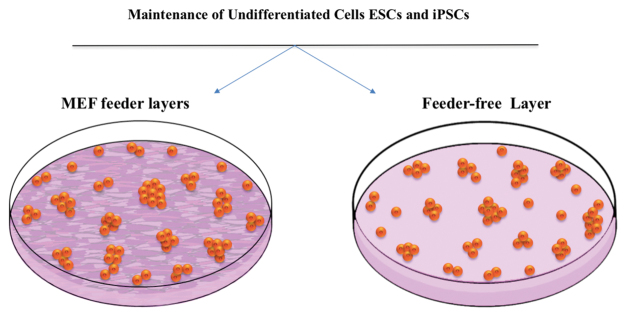
Coculture of ESCs or iPSCs on embryonic fibroblast feeder cell layers and feeder-free cell layers. Inactivated mouse and human-derived cells are traditionally utilized as feeder layers to retain the pluripotency of ESCs and iPSCs. Recently, a plethora of feeder-free layer systems was generated such as Matrigel^™^, gelatin-coated substrates, and iMatrix-511 to maintain ESCs and iPSCs in undifferentiated state for long-term culture.

A key concern in pluripotent stem cell related research is to maintain the pluripotent cultures in an undifferentiated and proliferative condition without causing chromosomal aberrations.^101^ To overcome this obstacle, the generated iPSCs are commonly maintained on mouse embryonic fibroblasts (MEFs) known as mouse feeder cells that secrete several unknown protein factors, which provide an optimal microenvironment for the iPSCs to sustain pluripotency (Fig. 5). MEFs in a fetal bovine serum-containing medium are traditionally used for ESC culture.^55,102^ However, the exposure of iPSCs to the feeder cells and their supplementary unidentified animal proteins can present a risk of xeno-contamination.^103^ Subsequently, Matrigel^™^ has been widely used as one of the feeder-free strategies to maintain ESCs for long-term culture,^104^ for cell cultivation and differentiation.^105^ However, Matrigel is isolated from Engelbreth-Holm-Swarm mouse tumor, which expresses lot-to-lot variations and can be a source of xenogeneic contaminants.^106^ Therefore, total animal substance removal and the use of serum-free medium are required to comply with the Standard for Biological Ingredients (SBI).

The use of gelatin with serum free condition could rapidly and steadily produce the ES-like cells.^103^ However, Haque et al.^107^ and Yamasaki et al.^103^ reported that the iPSCs grown in this condition could not retain their pluripotency without undergoing differentiation when cultured on gelatin surface. The reason for this phenomenon remains unknown. Perhaps, it was due to the choice of culture medium or to the type and concentration of serum used. However, a gelatin-based feeder-free culture system has been successfully used to maintain undifferentiated human pluripotent stem cells.^108,109^ The gelatin-based culture was also used in our laboratory for iPSC production and to maintain the self-renewal property of mouse iPSCs.^110^

Yamanaka's group has successfully developed StemFit^™^ medium utilizing recombinant laminin-511E8 cell surface (known as the iMatrix-511 system) as a novel culture system with high efficiency for self-renewal of both human ESCs and human iPSCs.^111^ Unfortunately, large-scale production and purification of biologically functioning recombinant laminins are laborious and costly.^105,112,113^ Besides, the expression and secretion of these factors can cause inconsistent generation of supportive cell layers. In addition, it is challenging to demonstrate which indispensable laminin isoforms support the pluripotent cell maintenance, as different isoforms of laminin exist.^105,114,115^

The use of culture medium supplemented with histone deacetylase inhibitor and transforming growth factor-β inhibitors during cellular conversion has been shown to improve the generation of iPSCs in the absence of either c-Myc or Klf4 and also substitutes Oct4 for the maintenance of pluripotency.^116–119^ It must be noted that reprogramming process under hypoxic conditions of 5% O_2_ with co-treatment of valproic acid (VPA) has a great influence on the reprogramming efficiency of both mouse and human cells.^120^ The reduced levels of O_2_ in cultures have positively contributed to the survival of neural crest cells^121^ and hematopoietic stem cells,^122^ while preventing human ESCs toward differentiation.^123^ Furthermore, it was reported that a soluble Wnt3a directly enhances the induction of pluripotency in the absence of exogenous c-Myc transduction.^124^

There are other signaling molecules which have a major contribution in the maintenance of mouse pluripotent state such as leukemia inhibitory factor (LIF is necessary for pluripotency *in vitro* only)^12^ and Activin/Nodal pathway,^125^ while fibroblast growth factor (FGF) and insulin-like growth factor (IGF) are sufficient to support the pluripotency of human pluripotent cells. Subsequently, the use of knockout serum replacement (KSR) has been shown to increase the number of colonies of AP-positive cells^126^ in the early event of reprogramming phase and also accelerates Oct4 expression during the late reprogramming phase.^127^ In addition, Liu et al.^128^ reported that Nanog expression was greatly enhanced when KSR-based medium was used to reprogram mouse fibroblasts.

## iPSCs in Cell Replacement Therapy

Besides disease modeling and drug discovery, one of the greatest potentials of iPSCs is in cell and gene replacement therapy for many genetic and degenerative diseases.^129,130^ In this approach, the somatic cells from a patient with disease are isolated and cultured. The cells are reprogrammed to iPSCs through viral or nonviral mediated gene transfer before the replacement of the disease-causing gene with a healthy gene. The genetically modified iPSCs are enriched and then subsequently differentiated into the affected cell subtype. The cells are then reinfused into the patient. This autologous transplantation approach may prevent serious complication such as graft-versus-host diseases, which commonly occur after allogeneic transplantation.

The iPSC application in cell replacement therapy became more evidenced few years following the first report by Yamanaka.^131^ In a cell replacement therapy for genetic disease, Raya et al.^132^ showed the production of phenotypically normal myeloid and erythroid lineages from iPSCs of a patient with Fanconi anemia. Subsequently, Ye et al.^133^ successfully demonstrated that β-thalassemia patient could synthesize healthy hemoglobin after autologous transplantation of genetically corrected hematopoietic stem cells derived from iPSCs.

In an attempt for cell replacement therapy for neurodegenerative diseases, Ebert et al.^24^ generated the very first motor neurons differentiated from the diseased iPSC, in which the iPSC was derived from a patient's skin fibroblast with spinal muscular atrophy. The degeneration of motor neurons was caused by loss-of-function mutations in the *SMN1* (survival of motor neuron 1, telomeric) gene. Motor neurons differentiated from the diseased iPSCs maintained the disease phenotype and could be partially alleviated by treatment with VPA and tobramycin, validating that iPSC can serve as a disease model for the development of new therapeutic strategies against degenerative disease.^130^

Liu et al.^134^ produced iPSCs from the smooth muscle of a Hutchinson-Gilford progeria syndrome (HGPS) patient exhibiting premature ageing and progressive reduction in the function of vascular smooth muscle. The diseased iPSCs from this patient exhibited cellular senescence compared to normal iPSCs. The mutated Lamin (A) locus was then corrected using homologous recombination of a helper-dependent adenoviral vector. These proof-of-principle studies have shown that the iPSC technology holds a huge potential in regenerative medicine in the near future.

In addition, the potentials of iPSC-derived CMs (iPSC-CMs) in therapy have also been explored *in vivo*. The iPSC-CMs transplanted into heterozygous monkeys with a major histocompatibility complex haplotype (HT4) subjected to myocardial infarction have improved cardiac contractile function at 4 and 12 weeks after transplantation. This demonstrates that the allogeneic iPSC-CM transplantation is sufficient to regenerate the infarcted primate heart, although subsequent arrhythmias have been observed.^135^

The first iPSC clinical trial (phase I) was conducted in September 2014 in Japan, less than a decade following the first generation of iPSCs. Masayo Takahashi from RIKEN Center of Developmental Biology demonstrated the safety of iPSC-based therapies for retinitis pigmentosa (RP), which is primarily characterized by extra blood vessel formation in the eye leading to a total loss of vision. One-year postoperation, the patient's eyesight has improved after receiving autologous photoreceptor sheets.^136^ In contrast, Kuriyan et al.^137^ reported a disastrous result for age-related macular degeneration (AMD) therapy using autologous adipose stem cells, whereby the patients had rapid loss of vision and needed emergency care. These events highlight an urgent need to establishing iPSC therapy with improved clinical outcomes.

Allogeneic transplant of human ESCs has been utilized to treat spinal cord injury in 2010,^138^ dry AMD in 2016,^139,140^ and type I diabetes mellitus in 2014.^141,142^ In these trials, the engraftment was highly efficient, and the treated individuals did not show signs of oncogenesis. However, allogeneic stem cell transplantation can be used solely in immune privileged sites, such as eyes and spinal cords.^143^ Personalized iPSCs may address the immune-related issues, but the generation and characterization of high-grade clinical iPSCs are cost intensive and time consuming.^143^

The establishment of iPSC bank, which is able to stockpile a large number of human leukocyte antigen (HLA)-homozygous super-donor iPSCs, is needed to overcome these issues.^143^ One such example is the Center for iPS Cell Research and Application (CiRA) at Kyoto University, which was established in 2013. CiRA is an allogeneic iPSC bank collecting 50 specimens of peripheral blood T lymphocytes and umbilical cord blood from healthy volunteers with homozygous HLA, matching at three major loci of HLA (HLA-A, HLA-B, and-DRB1). The collection is expected to cover 30–50% of Japanese population use in 2020.^144^ However, a higher number of donors (estimated 150) with homozygous HLA typed is needed to match 93% of the U.K. population.^145^ In 2015, Cellular Dynamic International, Inc., has also established a bank for cell therapy that can match 19% of U.S. population. It is believed that the banking of HLA-haplotype homozygous iPSCs that serve as an extensive library of appropriate cells for millions of recipients at affordable costs could be an effective strategy. It is noteworthy to point that the iPSC bank-acquired allogeneic iPSC-based clinical trials are ongoing to treat patients with cardiac failure, retinal pigment epithelium, and Parkinson's disease in Japan.^146^

Despite having tremendous therapeutic potential, the translational research of iPSC replacement therapy to human patients is relatively slow, highlighting the urgency for improved iPSC-based cell therapy. Due to the extremely low efficiency of homologous recombination in iPSCs,^147^ targeted genome editing could potentially overcome this limitation. Targeted genome editing is broadly applicable to genetically engineer any sequence of interest in living cells or organisms.^148,149^ Therefore, the combination of novel approaches in human iPSCs and CRISPR-based genome editing can improve iPSC-based cell therapy and create a viable option for stem cell therapy and regenerative medicine.

## CRISPR Editing of iPSCs

The bacterial CRISPR was first identified as a system of adaptive immunity against invading pathogens.^150,151^ In 2013, the CRISPR/Cas9 system was repurposed as a simple platform for genetic editing to generate site-specific nucleases through the use of the RNA-guided CRISPR-associated 9 (Cas9) proteins.^148,152^ CRISPR/Cas9 genome editing technology can either be used to generate a gene knockout by the deletion of the faulty gene, gene repair using homologous chromosome, or gene knock-in by introducing exogenous healthy gene to replace or augment a defective mutant gene.^153^

**FIG. 6.**
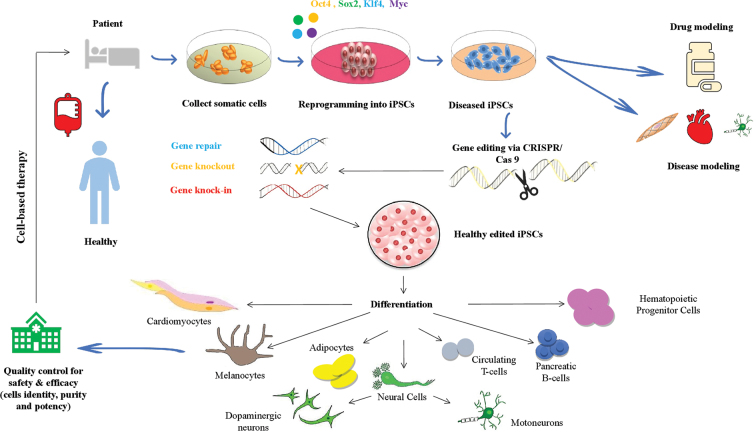
CRISPR/Cas9-mediated gene editing in the iPSCs derived from patient's somatic cells for cell replacement therapy to treat various genetic and degenerative diseases. Somatic cells isolated from a patient carrying mutation are reprogrammed into iPSCs by the introduction of Oct4, Sox2, Klf4, and c-Myc using either viral or nonviral gene transfer. The iPSCs are then genetically engineered to correct the mutation by the CRISPR/Cas9 technology. The corrected iPSCs are enriched and induced to differentiate into the target cells. Finally, the cells are reinfused into the patient to correct the disease condition. Cas9, CRISPR-associated 9; CRISPR, clustered regularly interspaced short palindromic repeats.

A plethora of studies have revealed that the CRISPR/Cas9 system can be used to reprogram somatic cells into iPSCs and to modify pluripotent cells genetically.^152–159^ Furthermore, a number of scientific reports confirm that the CRISPR/Cas9 genome editing and human iPSCs are two impactful tools for *in vitro* human disease modeling. This is by the creation of isogenic cellular materials, which are a precise control for a genetic disease model of interest to highlight phenotypic differences and also to elucidate pathological mechanism of complex diseases such as facial anomalies syndrome,^160^ Barth syndrome,^161^ RP,^162^ severe combined immunodeficiency,^163^ and spinocerebellar ataxia type 2 (SCA2).^164^ Moreover, using CRISPR/Cas9-mediated gene correction in patient-derived iPSCs is a potentially promising therapeutic intervention to cure genetic diseases such as ß-thalassemia,^165^ hemophilia A,^166^ sickle cell disease,^167^ cystic fibrosis,^168^ Duchenne muscular dystrophy,^169^ and hereditary deafness.^170^ In a landmark *ex vivo* study, CRISPR/Cas9 has been used to successfully correct a mutation in the hemoglobin beta (*HBB*) gene of iPSC clones from beta-thalassemia patient. The corrected cells with functioning *HBB* gene expression were subsequently differentiated into erythroblasts.^165^ Similarly, trinucleotide repeat (CAG) in Huntington gene (*HTT*) expression was corrected using CRISPR/Cas9 in iPSC neurons obtained from a Huntington's patient. The corrected cells with no phenotypic abnormalities were differentiated into synaptically active neurons.^171^ The CRISPR/Cas9-mediated gene editing in the iPSCs derived from patient's somatic cells for cell replacement therapy to treat various genetic and degenerative diseases is shown in Figure 6.

One of the main goals of iPSCs and CRISPR/Cas9 technology is to establish an iPSC bank to match the HLA phenotype diversity or to generate an iPSC “universal donor.” Recently Hotta and Kaneko laboratories have edited the major histocompatibility gene of iPSCs to eliminate immune rejection from both killer T cells and nature killer (NK) cells of recipient. In the first strategy, they created pseudo-HLA homozygous (or HLA haploid) iPSCs from HLA class I heterozygous healthy donors with precision-allele specific gene editing. In the second strategy, they utilized multiplexed gene editing technology to disrupt (MHC class I) HLA-A, and HLA-B bi-allelically, but retained a single haplotype of HLA-C allele (named, HLA-C retained iPSCs). This is to match the donor and also to maintain their antigen presentations that are important for suppressing the function of NK cells. The modifications not only enable the HLA-C retained iPSCs to elude T cells (CD8) but also to avoid NK cell activities *in vitro* and *in vivo*.^172^ In addition, the group also deleted MHC class II transactivator gene (CIITA) from the HLA-C retained iPSCs to evade HLA-DR-activated CD helper T cell toxicity, as a better option for donor–host matching.^172^ These cells are referred to as MHC-edited cells. Unfortunately, the presence of minor histocompatibility antigens (MiHA; also known as polymorphic peptides) on MHC-edited cells might trigger snap responses in patients, leading to shortage in the T cell response repertoire.^173^ Importantly, the safety of MHC-edited cells is absolutely a daunting pitfall and remains to be examined cautiously in clinical studies, as inbred animal models are unable to adequately replicate the adverse elasticity of human immune systems.^173^ Nevertheless, we believe that extreme caution is necessary before using such technology to human considering the devastating risks of tumorigenicity, off-target mutagenesis, and CRISPR/Cas9 targeting efficiency.

## Therapeutic Genome Editing

Chimeric antigen receptor (CAR) T cell therapeutic strategy has been shown to successfully mediate regression of hematologic malignancies.^174^ Unfortunately, engineered autologous T cell transplantation can be a burden to manufacture, particularly due to low lymphocyte counts and inefficient expansion of healthy T lymphocytes *in vitro*, owing to immunosuppression or previous cytotoxic treatment.^175^ The solution is to engineer patient-derived iPSCs to simultaneously produce antigen-specific CAR-based T cells with HLA-independent customizable antigen recognition.^175–177^ The genome-edited identical master human iPSCs can be further differentiated into fully functional histocompatible tumor-targeting T cells accessible to all patients regardless of their HLA haplotype.^178^

The potential benefit of iPSC derived CAR-engineered T (iCART) cells to kill cancer cells highlights a significant milestone toward clinical evolution and therapeutic usage. Thus far, pre-clinical studies in animal models have revealed that iCART cells have the potency as an antitumor activity.^179^ In 2019, the CiRA and Takeda collaboration have stated that the first iCART cell treatment program is due to commence for a clinical trial Phase I in 2021. The iCART treatment can be tailor-made to produce off-the-shelf immunotherapies for patients on demand.^180^

In contrast to T cells, NK cells are lymphocytes that play a pivotal role in the innate immune system's ability to mediate antimalignant and antiviral activities without requiring MHC restriction.^181^ Clinical trials utilizing NK cell-based immunotherapy have demonstrated remarkable efficacy against acute myeloid leukemia and less activity against other malignancies.^181^ However, as with T cells, healthy population of NK cells is difficult to obtain and is usually composed of a heterogeneous mixture of monocytes and other blood cells. The advent of iPSCs provides a unique solution to produce homogeneous and defined groups of NK cells that can be easily modified genetically with improved antitumor activity for clinical scale production of “off-the-shelf” cell-based therapy.^182,183^ The modified NK cells can express a variety of receptors such as high-affinity DC16 Fc receptor or CAR receptors or they can be combined with other therapies to improve their potency against solid tumors.^184–189^ So far, the first study showing the feasibility and efficacy of iPSCs as a platform to produce NK cells bearing cancer-homing CAR receptor (CARiPSC-NK cells) has shown impressive antitumor activity in an ovarian cancer xenograft model. The study also indicated cell survival expansion *in vivo* with less toxicity.^190^

The potential to transform these pre-clinical studies into clinical trials is exciting. In November 2018, the human clinical trial (NCT03841110) received the Food and Drug Administration (FDA) approval for the first use of its kind off-the-shelf NK cell produced from clonal master iPSCs (named FT500) synergized with T cells to better treat solid tumor malignancy. Upon administration to three patients (the first doses of FT500 with 1 × 10^8^ cells per dose combined with checkpoint inhibitors and monoclonal antibodies), the initial safety assessment showed no serious adverse events during the initial 28-day observation period. In February 2019, the FT500 therapy was further tested for safety in 64 patients with diverse malignancies. The clinical trial is currently in the beginning phases with an estimated result completion date of June 2020 (Clinicaltrials.gov Identifier: NCT03841110).

## Conclusion and Future Prospects

The human iPSCs present a uniquely scalable platform for the study of inherited diseases, cell modeling, and as a novel mean for cell replacement in clinical applications; thereby, replacing the controversial use of human ESCs. The advancements in human iPSCs have tremendous impact in regenerative medicine, particularly after the initial success of Japanese clinical trial in 2014. Remarkable achievements have been made in iPSC-based clinical trials for the past 13 years. The menace of tumorigenicity appears to be well controlled, while precise differentiation of iPSCs toward specific cell types for cell therapy products under GMP guidelines is achievable. This high-potential achievement is further enhanced when combined with genome engineering technology, such as the CRISPR/Cas9. This provides a potent strategy to correct mutations in patient-derived iPSCs, to modify lineage-specific reporter lines to facilitate differentiation toward a particular cell type, and to generate safe master iPSC clones as a resource for “off-the-shelf” cellular products. Although the field of iPSCs is still in its infancy and with significant risks, we are optimistic that iPSC application will offer a huge prospect in personalized regenerative medicine and cancer immunotherapy in the next decade.

## Abbreviations Used

AMD: age-related macular degeneration
AP: alkaline phosphatase
CAR: chimeric antigen receptor
Cas9: CRISPR-associated 9
CiRA: Center for iPS Cell Research and Application
CM: cardiomyocyte
CRISPR: *clustered regularly interspaced short palindromic repeats*
ESCs: embryonic stem cells
*HBB*: hemoglobin beta
iCART: iPSC derived CAR-engineered T
iPSCs: induced pluripotent stem cells
Klf4: Krüppel-like factor 4
KSR: knockout serum replacement
LV: lentiviral vector
MEFs: mouse embryonic fibroblasts
MET: mesenchymal-to-epithelial transition
miRNA: microRNA
NK: nature killer
Oct4: octamer-binding transcription factor 4
OSKM: Oct3/4, Sox2, Klf4, and c-Myc
PB: piggyBac
RP: retinitis pigmentosa
Se-V: Sendai-virus
Sox2: SRY (sex determining region Y)-box 2
VPA: valproic acid
